# Developing a Research Agenda for the Analysis of Product Supply: A Response to the Recent Commentaries

**DOI:** 10.34172/ijhpm.2020.25

**Published:** 2020-02-19

**Authors:** Raphael Lencucha, Anne Marie Thow

**Affiliations:** ^1^School of Physical and Occupational Therapy, McGill University, Montreal, QC, Canada.; ^2^Menzies Centre for Health Policy, Charles Perkins Centre, University of Sydney, Sydney, NSW, Australia.

## Introduction

We read the responses to our paper “*How Neoliberalism Is Shaping the Supply of Unhealthy Commodities and What This Means for NCD Prevention*”^1^ with great interest. The different disciplinary perspectives expanded and deepened our analysis of the relationship between neoliberalism and the supply of unhealthy commodities. Our effort to articulate the role of ideas, specifically the category of ideas associated with the neoliberal paradigm, and the relationship to institutions that in turn shape product supply, is to point to one aspect of the system that is often neglected, that of policy paradigms. Adding analysis of the power of economic elites and commercial interests, the need for consideration of locality, particularity and situating products in the political economy that generates them, the explicit consideration of alternative economic paradigms designed to achieve societal (rather than narrowly defined economic) goals, and conceptualization of actors and institutions within complex adaptative systems serves as a protection from reductionist thinking. This aligns with our own recognition that the neoliberal paradigm itself is one of a number of other factors including the power of economic elites and commercial interests to mobilize strategies that shape policy and public perception.

## Power, Elites and Influence

Labonté^2^ and Smith^3^ draw attention to the power of both industry and economic elites in shaping the relationship between government and market and the consumer environment. Labonté provides a compelling narrative of the transformation and cementing of neoliberal ideology over time in forms that serve the interests of global elites and capital accumulation.^2^ Smith also notes the need to attend to the relationship between power and structure,^3^ referring to the structures that result from the organizing principles of a neoliberal paradigm. An emphasis on economic elites and the structures that induce capital accumulation is critically important. We would add a note of caution, similar to Herrick’s critique of our own analysis, that among critical health scholars there is a tendency to reduce the problems of product supply to corporate power to the exclusion of other factors that shape the societal project of production and consumption. The ideational basis of this project requires attention, namely, the ideas and values that underpin consensus and contestation when it comes to the ‘right’ and the ‘good’ of economic production and health.^4^

We attempted to draw attention to the ways that ideas operate to organize action or inaction on product supply, as part of broader dynamic of factors at play, as power is mobilized and represented through different forms argumentation or the ability to mobilize the voice of publics around particular ideas.^5^ Townsend^6^ expands on this emphasis in her exploration of reframing the problem of non-communicable diseases in economic terms, suggesting that ideas or frames have power to persuade. There is certainly a need to build from this premise with nuanced and robust analysis. A constructivist approach to power sees the construct not simply as a tool wielded by the economic elite but a set of approaches that involve knowledge claims, facts, norms, ideas, and of course material resources mobilized by different actors to shape the institutional and decision-making context. From this perspective institutions are sites of contestation where ideas do indeed become entrenched or solidified in structures (namely the rules and resources that impact how something is governed) but are also subject to disruption and change. It is this disruption and change that can serve as a locus of inquiry. Namely, to ask what factors create disruption and how does this disruption lead to more permanent changes in the institutional environment? Perhaps this is where the theoretical work of sociology, and particularly the extensive ongoing debate about the relationship between structure and agency, can inform these lines of inquiry as noted by Lee and Crosbie,^7^ Smith^3^ and Battams.^8^ This emphasis on structure and agency, and within this relationship the expressions of power, is certainly complimentary to our desire to draw greater attention to the way that ideas, and specifically the structuring influence of the category of ideas that fall within a neoliberal paradigm, are both shaped by actors and subsequently mobilized, both implicitly and explicitly, by actors to shape the policy environment.

## Locating Structure and Agency in Time and Place

Lee and Crosbie,^7^ and Labonté,^2^ Smith^3^ and Herrick^9^ provide an important critique of the way we present the neoliberal paradigm in a somewhat ahistorical and reductionistic manner. We agree that in order to gain a fuller understanding of the way that the category of neoliberal ideas and corresponding policies have shaped product supply it important to attend to the locally and historically situated ways that neoliberalism has emerged and has been applied in different contexts. However, neoliberalism, despite being applied in varied ways and representing varied concepts of the relationship between state, market and society, has captured the imagination and mandates of powerful institutions.^10,11^ The hegemonic dominance of this paradigm is reflected, for example, in the rules that have been instituted in international trade and investment regimes.^12^ The emergence of institutions like the World Trade Organization illustrate both the centralization and diffusion of a common set of norms. The dominance of this category of ideas in the structuring of international economic institutions over the past half-decade does not eliminate the recognition that different principles of this paradigm have been applied in different forms in different contexts, and in many ways, this is the logical ‘next step’ of our reframing, in terms of serving as the basis for detailed analysis in specific contexts. In fact, as we noted, the significant wins for tobacco control measures against government and industry opposition in trade and investment fora indicates that there exists policy space in these regimes for government protection of health over corporate rights.^13,14^ Although we also recognize that such regimes are not uniformly implemented or uncontested by states, particularly with the persistent relevance of the contrary norms of sovereignty, national self-interest, and economic protectionism, the enduring relevance of the core principles of the neoliberal paradigm remain omnipresent in the imagination of policy-makers and the institutions in which they operate.^15-17^ We certainly do agree with Herrick, Lee and Crosbie and Labonté, that our paper treated neoliberalism in an overly uniform manner. We do hope that our overview of the generally accepted principles that informed the original conceptions of the neoliberal project provide starting points to understand the linkages between this paradigm and common contemporary approaches, albeit differentiated, taken by governments to product supply.

## Looking to New Paradigms

Schram and Goldman^18^ provide a refreshing consideration of alternative modes of production, making the point that “By underpinning market exchanges with food’s inherent value to our well-being, community life and environmental stewardship, alternative food economies are challenging neoliberalism’s conception of goods and services as ‘neutral,’ recognising value not captured in current supply, demand and price relations.” They provide starting points to consider alternative approaches to what is often implicitly accepted as the norms of production. This type of reimagining is an essential complement to fill the void that is inevitably left from critical deconstructions of dominant economic paradigms and their shortcomings.

This pursuit for alternative models of economics converges with wider efforts to bring together various sectors and actors in order to construct comprehensive and sustainable modes of production and consumption. These questions touch on Herrick’s concern that our characterization of ‘product-based risk’ is disconnected from the social, political and commercial forces that shape patterns of consumption.^9^ The situated and integrated approach that Herrick argues for eschews the type of product exceptionalism that has often characterized tobacco control efforts,^19,20^ and can further expand the scope of pursuit beyond products to consider ecological and human-constructed systems. Milsom et al^21^ bring forward a similar point from a different angle, arguing that an analytic gaze focused on policy and institutions as dynamic systems is required to understand both stagnation and change in the realm of policy. This emphasis on systems provides methodological challenges inherent in analyzing a web of factors that cannot necessarily be reduced to simple cause and effect relationships. Although we agree that systems thinking can assist broader recognition that one must identify a web of factors over time to fully understand product supply, one risk with this approach is that the system itself becomes analytically diffuse. This is where a priori conceptual work is important in identifying an initial set of factors that are at least conceptually associated with the research question, which in this case is what factors shape governments approach to product supply.

## Conclusion

The commentaries offer an important reflection on future lines of inquiry to pursue the overarching question of what has shaped the approach taken by governments to product supply. Here we synthesise these lines of inquiry drawn from the eight commentaries, in order to guide future analyses.

First, there is the critical need to examine how institutions shape or mediate the influence of unhealthy commodity producing industries on public policy, and to interrogate the ways in which neoliberalism as a dominant paradigm has been translated into policy in agriculture, finance, trade and other domains of policy in different contexts. Further, it will be important to ask who is exercising power, and in what ways, in commodity-related policy-making, recognizing that power is not a zero-sum game with numerous tools and avenues to exercise power. In addition, the effects of power also need to be interrogated, by asking how commodity-producing industries have been treated by different governments, and different sectors within government, and how this treatment has shaped the supply of unhealthy (and healthy) commodities.

A second theme relates to operationalising an integrated approach to governance, which fills the space left by critical analysis of existing paradigms. This includes asking how current alternatives, such as the sustainable development paradigm, have (or haven’t) impacted ways of thinking about agricultural production as it pertains to health and consumer choice. Analyses of the governance of commodities can grant insights into potential implications of alternative economic paradigms. The development and analysis of positive cases will provide an important complement to the deconstruction of cases where paradigms lead to poor health outcomes.

Finally, it is essential that we ask how we can better theorize both structure and agency and the relationship between the two in order to inform the construction of policy and institutions that promote the supply of healthy commodities. Here we can look to the giants of sociology like Gidden’s structuration theory,^22^ or Archer’s morphogenetic approach^23^ that provides a critique of what she terms ‘conflationist’ approaches (including Gidden’s structuration theory) to the problem of structure and agency, or Foucault who deconstructs the embedded power within knowledge systems and how institutions generate and mobilize authority to shape society.^24^ This is certainly a project that requires the bridging of a robust social and political theory with empirical analysis of diverse contexts.

## Ethical issues

Not applicable.

## Competing interests

Authors declare that they have no competing interests.

## Authors’ contributions

RL completed the first draft of this manuscript. AMT made important revisions to the draft. RL and AMT revised subsequent drafts and approve the final version.

## Authors’ affiliations

^1^School of Physical and Occupational Therapy, McGill University, Montreal, QC, Canada. ^2^Menzies Centre for Health Policy, Charles Perkins Centre, University of Sydney, Sydney, NSW, Australia.

## References

[1] Lencucha R, Thow AM (2019). How neoliberalism is shaping the supply of unhealthy commodities and what this means for NCD prevention. Int J Health Policy Manag.

[2] Labonté R (2020). Neoliberalism 40: the rise of illiberal capitalism: Comment on “How neoliberalism is shaping the supply of unhealthy commodities and what this means for NCD prevention. Int J Health Policy Manag”.

[3] Smith J (2020). Towards critical analysis of the political determinants of health: Comment on “How neoliberalism is shaping the supply of unhealthy commodities and what this means for NCD prevention”. Int J Health Policy Manag.

[4] Benatar S, Upshur R, Gill S (2018). Understanding the relationship between ethics, neoliberalism and power as a step towards improving the health of people and our planet. Anthr Rev.

[5] Schmidt VA (2008). Discursive institutionalism: the explanatory power of ideas and discourse. Annu Rev Polit Sci.

[6] Townsend B (2020). Next steps for elevating health on trade and investment policy agendas: Comment on “How neoliberalism is shaping the supply of unhealthy commodities and what this means for NCD prevention”. Int J Health Policy Manag.

[7] Lee K, Crosbie E (2020). Understanding structure and agency as commercial determinants of health: Comment on “How neoliberalism is shaping the supply of unhealthy commodities and what this means for NCD prevention”. Int J Health Policy Manag.

[8] Battams S (2020). Neo-Liberalism, policy incoherence and discourse coalitions influencing non-communicable disease strategy: Comment on “How neoliberalism is shaping the supply of unhealthy commodities and what this means for NCD prevention”. Int J Health Policy Manag.

[9] Herrick C (2020). On the perils of universal and product-led thinking: Comment on “How neoliberalism is shaping the supply of unhealthy commodities and what this means for NCD prevention”. Int J Health Policy Manag.

[10] Harvey D (2007). A Brief History of Neoliberalism. Oxford University Press.

[11] Glasgow S, Schrecker T (2015). The double burden of neoliberalism? Noncommunicable disease policies and the global political economy of risk. Health Place.

[12] Lang A (2011). World Trade Law After Neoliberalism: Reimagining the Global Economic Order. Oxford University Press.

[13] Drope J, Lencucha R (2014). Evolving norms at the intersection of health and trade. J Health Polit Policy Law.

[14] McGrady B, Jones A (2013). Tobacco control and beyond: the broader implications of United States Clove Cigarettes for non-communicable diseases. Am J Law Med.

[15] Lencucha R, Reddy SK, Labonte R (2018). Global tobacco control and economic norms: an analysis of normative commitments in Kenya, Malawi and Zambia. Health Policy Plan.

[16] Labonté R, Stuckler D (2016). The rise of neoliberalism: how bad economics imperils health and what to do about it. J Epidemiol Community Health.

[17] Schrecker T (2016). ‘Neoliberal epidemics’ and public health: sometimes the world is less complicated than it appears. Crit Public Health.

[18] Schram A, Goldman S (2020). Paradigm shift: new ideas for a structural approach to NCD prevention: Comment on “How neoliberalism is shaping the supply of unhealthy commodities and what this means for NCD prevention”. Int J Health Policy Manag.

[19] Collin J (2012). Tobacco control, global health policy and development: towards policy coherence in global governance. Tob Control.

[20] McGrady B (2007). Trade liberalisation and tobacco control: moving from a policy of exclusion towards a more comprehensive policy. Tob Control.

[21] Milsom P, Smith R, Walls H (2020). A Systems thinking approach to inform coherent policy action for NCD prevention: Comment on “How neoliberalism is shaping the supply of unhealthy commodities and what this means for NCD prevention”. Int J Health Policy Manag.

[22] Giddens A (1984). The Constitution of Society: Outline of the Theory of Structuration. University of California Press.

[23] Archer MS. Realist Social Theory: The Morphogenetic Approach. Cambridge: Cambridge University Press; 1995.

[24] Hamann TH (2009). Neoliberalism, Governmentality, and Ethics. Foucault Stud.

